# Pre-contrast T1 and cartilage thickness as confounding factors in dGEMRIC when evaluating human cartilage adaptation to physical activity

**DOI:** 10.1186/s12880-019-0399-0

**Published:** 2019-12-31

**Authors:** Carl Johan Tiderius, Zana K. Hawezi, Lars E. Olsson, Leif E. Dahlberg

**Affiliations:** 1 Orthopaedics, Department of Clinical Sciences Lund, Lund University, Skåne University Hospital, Lund, Sweden; 2 Medical Radiation Physics, Department of Translational Medicine, Lund University, Malmö, Sweden; 3 Department of Haematology, Oncology and Radiation Physics, Skåne University Hospital, Malmö, Sweden

**Keywords:** dGEMRIC, Sub-regional analyses, Cartilage, Exercise

## Abstract

**Background:**

The dGEMRIC (delayed Gadolinium-Enhanced MRI of Cartilage) technique has been used in numerous studies for quantitative in vivo evaluation of the relative glycosaminoglycan (GAG) content in cartilage. The purpose of this study was to determine the influence of pre-contrast T1 and cartilage thickness when assessing knee joint cartilage quality with dGEMRIC.

**Methods:**

Cartilage thickness and T1 relaxation time were measured in the central part of the femoral condyles before and two hours after intravenous Gd-DTPA^2−^ administration in 17 healthy volunteers from a previous study divided into two groups: 9 sedentary volunteers and 8 exercising elite runners. Results were analyzed in superficial and a deep weight-bearing, as well as in non-weight-bearing regions of interest.

**Results:**

In the medial compartment, the cartilage was thicker in the exercising group, in weight-bearing and non-weight-bearing segments. In most of the segments, the T1 pre-contrast value was longer in the exercising group compared to the sedentary group. Both groups had a longer pre-contrast T1 in the superficial cartilage than in the deep cartilage. In the superficial cartilage, the gadolinium concentration was independent of cartilage thickness. In contrast, there was a linear correlation between the gadolinium concentration and cartilage thickness in the deep cartilage region.

**Conclusion:**

Cartilage pre-contrast T1 and thickness are sources of error in dGEMRIC that should be considered when analysing bulk values. Our results indicate that differences in cartilage structure due to exercise and weight-bearing may be less pronounced than previously demonstrated.

## Background

The dGEMRIC (delayed Gadolinium-Enhanced MRI of Cartilage) technique has been used in numerous studies for quantitative in vivo evaluation of the relative glycosaminoglycan (GAG) content in cartilage [1–6]. Most studies have analysed bulk values, i.e. a mean post-contrast T1 value in full-thickness cartilage regions of interest (ROI), also referred to as the “dGEMRIC index” [7]. Many studies support the notion that the dGEMRIC index adequately describes the cartilage quality in subjects with early osteoarthritis (OA), rheumatoid arthritis and hip dysplasia [8–10]. However, it has become obvious that there are confounding factors when using bulk values for GAG estimation. In particular, neither variations in the preT1 values nor differences in cartilage thickness are considered when analysing the dGEMRIC index [11].

Previously, it has been assumed that T1 of native cartilage is relatively constant between subjects [12], which has led to the conclusion that measuring pre-contrast T1 is not necessary. However, pre-contrast T1 in cartilage has shown a significant variation with depth [11, 13, 14]. In healthy volunteers, T1 decreases from 900 to 1100 ms in superficial cartilage to 400–500 ms in deep cartilage measured at 1.5 T. In addition, early stage OA has been associated with increased pre-contrast T1 values [11, 13–15].

The uncertainty of the dGEMRIC index is further complicated by the fact that cartilage thickness varies between subjects. At the time of imaging, often 90–120 min post injection, contrast medium penetration is more complete in a thinner cartilage than in a thicker cartilage [11].

Sub-regional dGEMRIC analysis has been suggested to address these issues [11, 13, 14]. However, in subjects with radiographic OA (i.e. reduced cartilage thickness caused by joint space narrowing), sub-regional dGEMRIC analysis did not show any difference in sensitivity compared to full-thickness analysis [16].

Previously, we found that elite runners had higher (bulk) dGEMRIC index than sedentary individuals [17] and concluded that this was likely due to a higher cartilage GAG content among elite runners. However, in that study using the dGEMRIC index, the preT1 values and cartilage thickness were not included in the calculations. To examine the validity of our conclusion, that cartilage quality differs between active and sedentary individuals, we here perform a detailed re-examination of the image data [17] by measuring cartilage thickness as well as analysing gadolinum concentration in separate superficial and deep cartilage regions.

## Methods

### Subjects

In this retrospective study we included 17 volunteers from a previous study of healthy subjects, i.e. no history of knee injury or knee surgery, with different levels of physical activity [17]. Included were those that had both pre- and post-contrast T1 images at the previous investigation. The included subjects were divided into the following two groups:

Group I (the sedentary group) consisted of 9 sedentary volunteers (two of which were females) with the following characteristics: mean age 24 (range 21–30) years, mean height 173 (range; 159–184) cm, mean weight 69 (range; 50–91) kg and mean body mass index (BMI) 23 (range; 17.3–26.9).

Group II (the exercising group) consisted of 8 male elite athlete runners, each running a distance of 70–90 km per week, with the following characteristics: mean age 25 (range; 23–29) years, mean height 182 (range; 175–188) cm, mean weight 71 (range; 69–76) kg, and BMI 21.4 (range; 20.4–23.2).

### Magnetic resonance imaging (MRI) measurements

The MRI examination in the previous study [17] was performed prior to and 2 h after intravenous injection of Gd-DTPA^2−^ (0.3 mmol/kg of Gd-DTPA^2−^, Magnevist, Bayer Healthcare Pharmaceuticals, Germany) in an 1.5 T system (Siemens Magnetom Vision) with a dedicated knee coil. For T1 analysis of articular cartilage, a set of 5–6 inversion recovery turbo spin echo images was used pre-contrast and post-contrast (Repetition time, TR = 2000 ms, echo time, TE = 15 ms, turbo factor 11, inversion time, TI between = 50, 100, 200, 400, 800 and 1600 ms, matrix = 256*256, Field of view, FOV = 120*120 mm^2^, in-plane resolution 0.47 mm, slice thickness = 3 mm). Two sagittal slices were localized to cover the central part of the lateral and medial femoral condyle, respectively. To maximize the contrast uptake, volunteers walked two levels of stairs up and down five times, corresponding to seven minutes of exercise, immediately after contrast agent injection.

### Image analysis

**Fig. 1:**
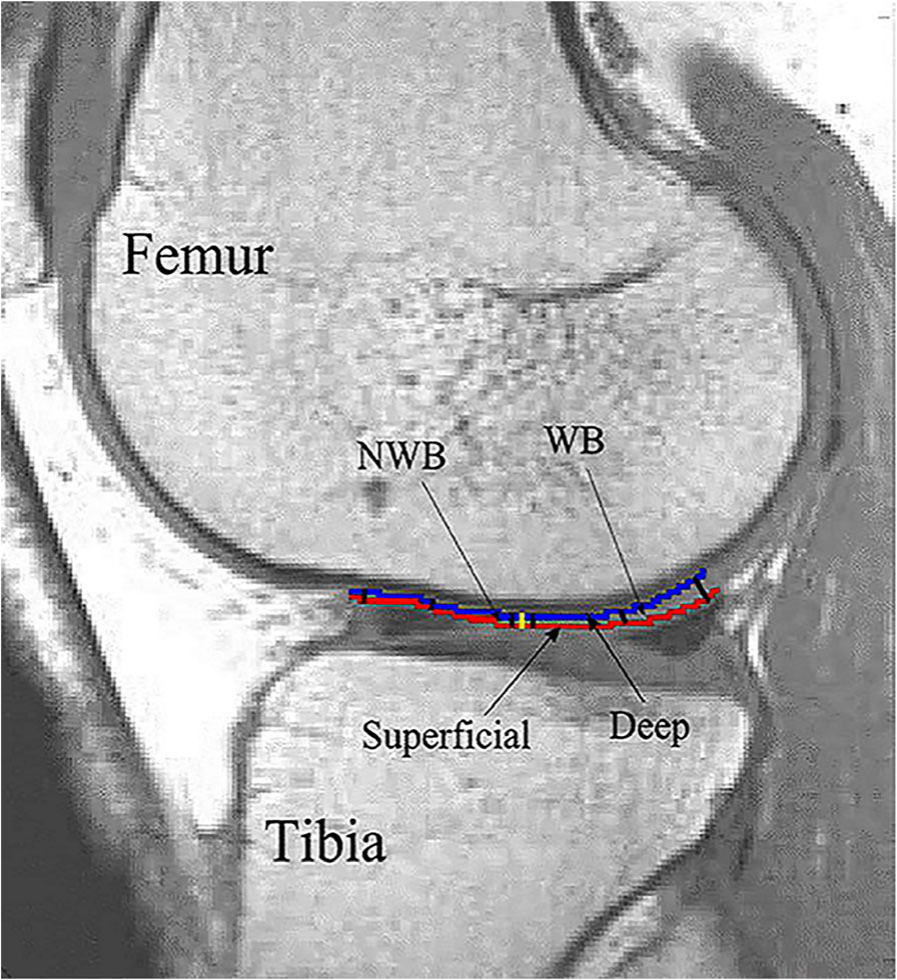
Illustration of the regions of interest (ROIs) in a lateral femoral condyle. The femoral knee cartilage was divided into two main segments: weight-bearing (WB) and non-weight-bearing (NWB). The deepest (blue) and the most superficial (red) cartilage layers of each segment were analysed as separate ROIs. The thickness of each segment was calculated as the mean value of three thickness measurements within each segment (indicated by black lines)

T_1_ calculations and segmentation of the ROIs were performed using MATLAB® (Mathworks Inc., Natick, MA). In the sagittal plane, the central parts of both the medial and lateral femoral condyles were divided into two main segments (weight-bearing, WB and non-weight-bearing, NWB) (Fig. 1) [18]. Identical location of the two sagittal slices (one in the medial and one in the lateral femoral compartment) in-between scans were carefully selected by the MRI technician. In addition, the first author (Z.H.) investigated that the thickness of the cartilage within each ROI did not change in-between repeated scans. Each segment was analysed as a full-thickness ROI (bulk value); the deepest and the most superficial cartilage layers (carefully avoiding extra-cartilage tissues) were also analysed as separate ROIs (Fig. 1). The segmentation procedure has been described in detail previously [11]. Pre- and post-contrast T1 values (post-contrast T1 = dGEMRIC index) for deep and superficial ROIs in each segment were computed. All dGEMRIC indices were corrected for BMI dosing-bias according to the formula recommended for cross-sectional studies, i.e. normalize the values to a BMI of 20 [19]:

$$ T{1}_{corrected}=T{1}_{measured}+3\ast \left( BMI-20\right)\kern0.50em \ (ms) $$

Estimated gadolinium concentrations for all regions of interest were calculated using the following formula:

[Gd] = (1/T1_Gd_ – 1/T1_pre_)/r_1_,

where T1_Gd_ is the T1 value after contrast agent injection, T1_pre_ is the T1 value before Gd-DTPA^2−^ injection, and r_1_ is the relaxivity of Gd-DTPA^2−^, for which the value 4.1 s^− 1^ mM^− 1^ measured in human plasma at 37 °C temperature was used [20].

### Cartilage thickness

Cartilage thickness was measured using the ImageJ program [21]. Three measurements in each segment of the femoral cartilage was performed and the mean value was calculated.

### Statistical analysis

Sigma Plot 11 was used for the statistical analyses. Wilcoxon signed rank test was used for the comparison between weight-bearing and non-weight-bearing segments within a single group (sedentary or exercise) and also between deep and superficial regions within a single segment of a group. Mann-Whitney rank sum test was used for the comparison between groups. A *p* value below 0.05 was considered statistically significant. To analyse the relationship between cartilage thickness and gadolinium concentration, and also to compare bulk dGEMRIC index and superficial gadolinium concentration, Spearman’s rank correlation coefficient was used.

## Results

The medial, but not the lateral, femoral cartilage was thicker in the exercising than in the sedentary group in weight- and non-weight-bearing segments (Table 1).

**Table 1:** Cartilage thickness in the medial and lateral femoral cartilage in the exercising and the sedentary groups (mm ± SD). WB denotes Weight-Bearing and NWB Non Weight-Bearing

	Cartilage thickness (mm) Exercise		Cartlilage thickness (mm) Sedentary		*P* value (exercise vs sedentary)
	Mean	SD	Mean	SD	
WB					
Medialt	2.90	0.30	1.70	0.50	*p* = 0.005
Lateralt	2.87	0.21	2.54	0.40	*p* = 0.2
NWB					
Medialt	1.81	0.19	1.54	0.20	*p* = 0.03
Lateralt	1.73	0.20	1.56	0.30	p = 0.1

In most of the segments, the T1 pre-contrast value was longer in the exercising compared to the sedentary group (Table 2). In the sedentary as well as the exercising groups, the T1 pre-contrast value was longer in the superficial region compared to the deep region in weight-bearing as well as in non-weight-bearing segments (Table 2).

**Table 2:** Pre-contrast T1 (ms) (mean and SD) in superficial and deep femoral knee cartilage in Non Weight-Bearing (NWB) and Weight-Bearing (WB) regions of interest in the exercise and the sedentary group

	Pre-contrast T1 (ms) Exercise		Pre-contrast T1 (ms) Sedentary		*p* value (exercise vs sedentary)
	Mean	SD	Mean	SD	
NWB					
Superficial	1084	83	1003	132	*p* = 0.02
Deep	737	83	703	91	*p* < 0.001
p value (superficial vs deep)	*p* < 0.0001		*p* < 0.0001		
WB					
Superficial	1141	59	1104	114	*p* = 0.004
Deep	627	84	673	69	*p* = 0.1
p value (superficial vs deep)	*p* < 0.0001		*p* < 0.0001		

**Fig. 2:**
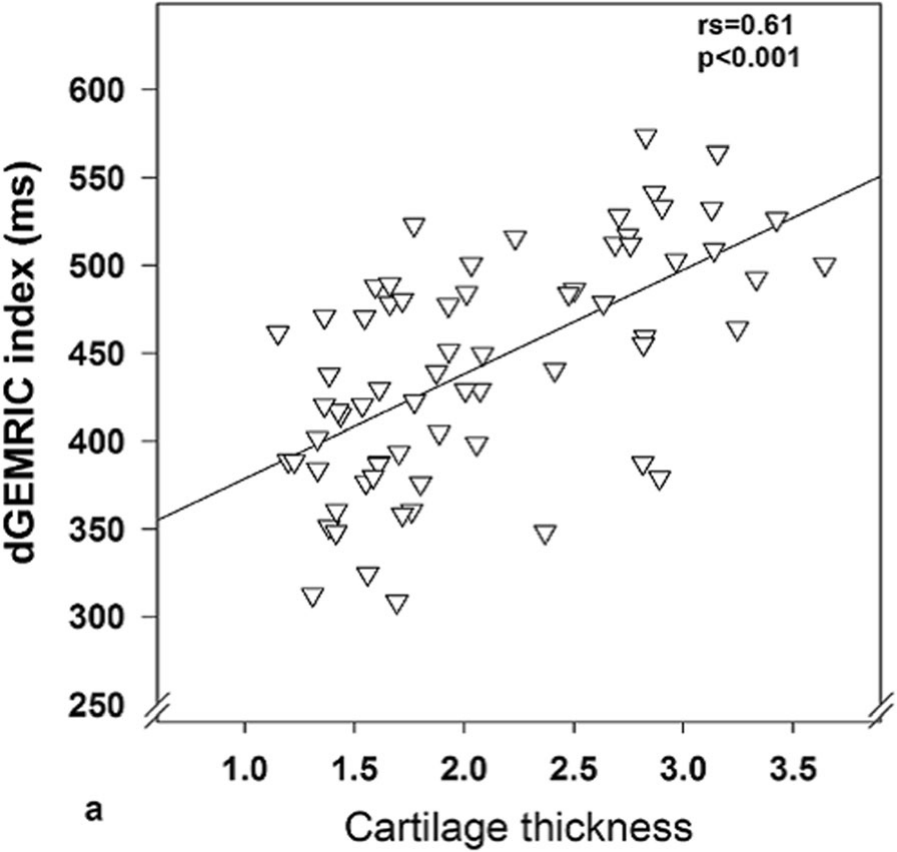
The correlation between cartilage thickness (mm) and dGEMRIC index (ms)

**Fig. 3:**
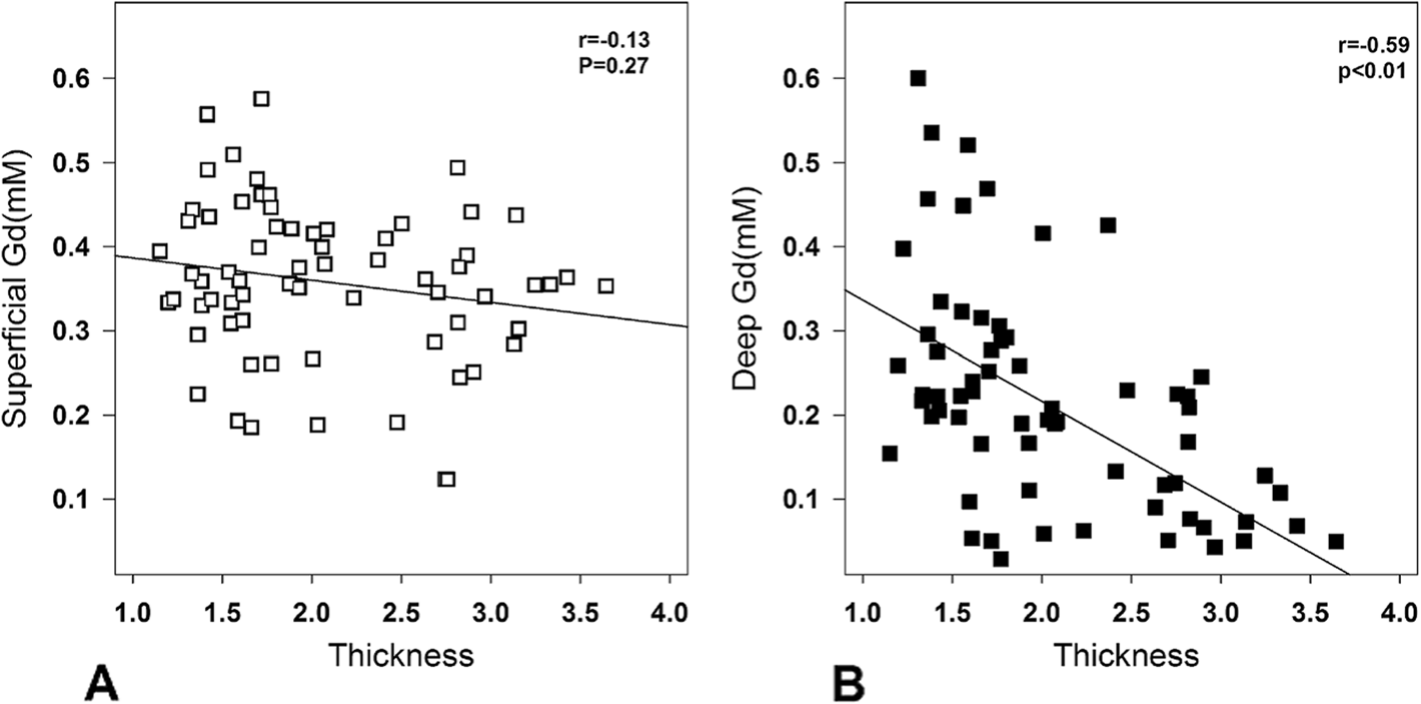
The correlation between the cartilage thickness (mm) and the gadolinium concentration (mM) in the superficial cartilage in all segmented ROIs (**a**) and in the deep cartilage in all segment ROIs (**b**)

The impact of cartilage thickness on the bulk dGEMRIC values is illustrated in Fig. 2 showing a significant correlation between thickness and index. Similarly, the gadolinium concentration was negatively related to cartilage thickness (Fig. 3b). In contrast, in superficial ROIs that were equally thick, the gadolinium concentration was not dependent on cartilage thickness (Fig. 3a).

The comparison between weight-bearing and non-weight bearing cartilage related to gadolinium concentration in superficial ROI shows a tendency to lower gadolinium concentrations in the weight-bearing compared to non-weight bearing segments in the exercise group (*p* = 0.07) but not the sedentary group (*p* = 0.3) (Table 3). However, there was no difference in the gadolinium concentration between exercising and sedentary individuals in the weight-bearing (*p* = 0.16) or in the non-weight-bearing segments (*p* = 0.5) (Table 3).

**Table 3:** Gadolinium concentration for the superficial region of interest for Non Weight-Bearing (NWB) and Weight-Bearing (WB) cartilage for exercise and sedentary groups, respectively

	Superficial [Gd] (mM) Exercise		Superficial [Gd] (mM) Sedentary)		*p* value (exercise vs sedentary)
	Mean	SD	Mean	SD	
NWB	0.37	0.08	0.39	0.07	*p* = 0.5
WB	0.29	0.08	0.35	0.09	*p* = 0.16
*p* value (NWB vs WB)	*p* = 0.07		*p* = 0.3		

## Discussion

In a previous dGEMRIC study using bulk (full-thickness) index values, we found higher dGEMRIC index interpreted as better cartilage quality in exercising compared to sedentary healthy volunteers [17]. To examine the impact of recently described confounding factors in dGEMRIC, we now have performed a detailed re-analysis of our data including T1 pre contrast and cartilage thickness measurements. Unexpectedly, T1 pre-contrast (native T1) was different for the exercise and sedentary groups; exercising individuals having longer native T1. This will influence the dGEMRIC index, which is the combined effect of native T1 and the shortening of T1 caused by the contrast agent. Hence, the exercising individuals in this study will get a higher dGEMRIC index, due to longer native T1 than sedentary individuals.

In the present study, we also confirm previous findings that the dGEMRIC index is dependent on cartilage thickness [11]. In that study, we also showed that the contrast medium mainly enters the cartilage from the surface and not via the subchondral bone [11]. Thus, particularly in subjects with thicker joint cartilage, the diffusion of the contrast medium into the deeper cartilage will be prolonged. This together with the fact that native cartilage in deeper parts has a T1 close to that of superficial cartilage diffused with gadolinium [11], do have an impact on the average bulk ROI value. Accordingly, both native T1 and cartilage thickness are confounding factors in cross-sectional dGEMRIC studies. For example, in this study the cartilage thickness and the pre-contrast T1 were different for the exercise and sedentary groups, even if it was only significant for the medial femoral cartilage.

The small number of subjects in this study is a power limitation in the statistical analysis. The trends of adaptation related to exercise and weight-bearing may had become significant with a larger number of subjects. In support of this contention, results from several experimental studies have shown cartilage adaptation to load and exercise [22–29], as well as two previous in vivo dGEMRIC studies [30, 31]. Due to the limited spatial resolution of the MR-images, it is challenging task to segment the different compartments of the cartilage. To reduce uncertainty all segmentation was performed by one single observer.

It may be argued that different gender may be a possible limitation in the present study. However, a previous meta-analysis has demonstrated that dGEMRIC results do not differ between men and women [32]. Another limitation is the T1 measurement protocol. Longer inversion times would have been more appropriate for the higher pre-contrast T1 in the cartilage. As a result, the uncertainty of the measured pre-contrast T1 is expected to be larger than for the post-contrast T1.

The study used triple dose (0.3 mmol/kg) of the contrast medium, while most studies use double dose. We have previously demonstrated a linear dose-response of gadolinium concentration in femoral knee cartilage after intravenous injection [33]. Therefore, we assume that the results in this study would have been similar after the double dose. However, triple dose may increase the sensitivity of the method [33].

Sub-regional analysis of cartilage has also been performed by others. In a study by Li et al. [16], sub-regional analysis was not more sensitive at identifying diseased cartilage than using bulk analysis, even though depth wise variations were clearly identified. Their result is somewhat contradictory to the result of this study. However, the definition of a deep ROI and a superficial ROI differs between our study and that study. In the study by Li et al., the cartilage was thinner due to OA resulting in a lack of superficial cartilage. To improve our understanding of human cartilage biology, it would be advantageous in future studies to examine diffusion and distribution patterns of contrast compounds at different stages of normal and degenerative cartilage, cartilage thickness and also including measures of physical activity and native cartilage T1.

## Conclusion

This study provides important information that exercising individuals have thicker cartilage and longer native T1 than sedentary individuals do. Both these factors influence post contrast T1 analysis, which complicates the interpretation of dGEMRIC bulk results.

Cartilage pre-contrast T1 and thickness are sources of error in dGEMRIC that should be considered when analysing bulk values. Our results indicate that differences in cartilage structure due to exercise and weight-bearing may be less pronounced than previously demonstrated.

## Data Availability

The datasets analysed during the current study are available from the corresponding author on reasonable request.

## Abbreviations

BMI: Body Mass Index
dGEMRIC: Delayed Gadolinium-Enhanced MRI of Cartilage
FOV: Field of View
GAG: Glycosaminoglycan
NWB: Non Weight-Bearing
OA: Osteoarthritis
ROI: Region of Interest
TE: Echo time
TI: Inversion time
TR: Repetition time
WB: Weight-Bearing (WB)
